# Empagliflozin suppressed cardiac fibrogenesis through sodium-hydrogen exchanger inhibition and modulation of the calcium homeostasis

**DOI:** 10.1186/s12933-023-01756-0

**Published:** 2023-02-06

**Authors:** Cheng-Chih Chung, Yung-Kuo Lin, Yao-Chang Chen, Yu-Hsun Kao, Yung-Hsin Yeh, Nguyen Ngoc Trang, Yi-Jen Chen

**Affiliations:** 1 Division of Cardiology, Department of Internal Medicine, School of Medicine, College of Medicine, Taipei Medical University, Taipei, Taiwan; 2 Division of Cardiovascular Medicine, Department of Internal Medicine, Wan Fang Hospital, Taipei Medical University, Taipei, Taiwan; 3 Taipei Heart Institute, Taipei Medical University, Taipei, Taiwan; 4 Department of Biomedical Engineering, National Defense Medical Center, Taipei, Taiwan; 5 Graduate Institute of Clinical Medicine, College of Medicine, Taipei Medical University, No. 250, Wu-Hsing Street, 11031 Taipei, Taiwan; 6 Department of Medical Education and Research, Wan Fang Hospital, Taipei Medical University, Taipei, Taiwan; 7 Division of Cardiology, Chang Gung Memorial Hospital, Taoyuan, Taiwan; 8 College of Medicine, Chang Gung University, Taoyuan, Taiwan; 9 Radiology Center, Bach Mai Hospital, Hanoi, Vietnam

**Keywords:** Fibroblasts, Fibrosis, Empagliflozin, Sodium-glucose co-transporter 2, Calcium, Sodium-Hydrogen exchanger

## Abstract

**Background:**

The novel sodium-glucose co-transporter 2 inhibitor (SGLT2i) potentially ameliorates heart failure and reduces cardiac arrhythmia. Cardiac fibrosis plays a pivotal role in the pathophysiology of HF and atrial myopathy, but the effect of SGLT2i on fibrogenesis remains to be elucidated. This study investigated whether SGLT2i directly modulates fibroblast activities and its underlying mechanisms.

**Methods and results:**

Migration, proliferation analyses, intracellular pH assay, intracellular inositol triphosphate (IP3) assay, Ca^2+^ fluorescence imaging, and Western blotting were applied to human atrial fibroblasts. Empagliflozin (an SGLT2i, 1, or 5 μmol/L) reduced migration capability and collagen type I, and III production. Compared with control cells, empagliflozin (1 μmol/L)- treated atrial fibroblasts exhibited lower endoplasmic reticulum (ER) Ca^2+^ leakage, Ca^2+^ entry, inositol trisphosphate (IP3), lower expression of phosphorylated phospholipase C (PLC), and lower intracellular pH. In the presence of cariporide (an Na^+^-H^+^ exchanger (NHE) inhibitor, 10 μmol/L), control and empagliflozin (1 μmol/L)-treated atrial fibroblasts revealed similar intracellular pH, ER Ca^2+^ leakage, Ca^2+^ entry, phosphorylated PLC, pro-collagen type I, type III protein expression, and migration capability. Moreover, empagliflozin (10 mg/kg/day orally for 28 consecutive days) significantly increased left ventricle systolic function, ß-hydroxybutyrate and decreased atrial fibrosis, in isoproterenol (100 mg/kg, subcutaneous injection)-induced HF rats.

**Conclusions:**

By inhibiting NHE, empagliflozin decreases the expression of phosphorylated PLC and IP3 production, thereby reducing ER Ca^2+^ release, extracellular Ca^2+^ entry and the profibrotic activities of atrial fibroblasts.

**Supplementary Information:**

The online version contains supplementary material available at 10.1186/s12933-023-01756-0.

## Introduction

Sodium-glucose co-transporter 2 inhibitors (SGLT2i) are novel class of anti-diabetic agents that reduce the risk of cardiovascular death and hospitalization in patients with heart failure (HF) and type 2 diabetes [1, 2]. SGLT2i may reduce cardiac fibrosis and improve cardiac function [3, 4]. Atrial fibrosis is a distinct and critical characteristic of atrial myopathy and atrial arrhythmogenesis [5, 6]. Patients with HF exhibit a higher incidence of atrial fibrosis and SGLT2i reduces left atrial filling pressure and increases exercise tolerance and diastolic function, all of which are correlated with atrial fibrosis [7–12]. However, whether and how SGLT2i may modulate atrial fibrogenesis remain unclear.

The calcium (Ca^2+^) signaling pathway plays a critical role in fibrogenesis and induces proliferation, collagen production, migration, and myofibroblast differentiation capabilities of fibroblasts [13–16]. SGLT2i can directly interact with the extracellular Na^+^ binding site of the Na^+^/H^+^ exchanger (NHE) thereby decreasing NHE activity and lowering the intracellular pH [17].

Increasing intracellular pH has been proven to induce cytosolic Ca^2+^ through endoplasmic reticulum (ER) Ca^2+^ leakage or Ca^2+^ influx [18, 19]. NHE activity is upregulated in patients with HF [20, 21] and NHE inhibition by cariporide decreases cardiac fibrosis in HF [22–24]. Increased NHE1 activity increases intracellular pH and activates cell migration capability [21, 25]. Dapagliflozin attenuates NHE1 gene expression [26]. Accordingly, SGLT2i may directly suppress fibrogenesis by inhibiting NHE signaling, leading to anti-fibrosis potential. The purpose of this study was to examine whether empagliflozin, an SGLT2i may decrease atrial fibrogenesis and study its underlying mechanisms.

## Materials and methods

### Cell cultures

Human atrial fibroblasts were purchased from Lonza Research Laboratory (Walkersville, MD, USA). The fibroblasts were seeded on uncoated culture dishes as monolayers in FGM™-3 Cardiac Fibroblast Growth Medium-3 BulletKit (Lonza, including HEPES: 14.999 mmol/L and sodium bicarbonate: 14.010 mmol/L) at 37 °C with 5% CO_2_. Cells from passages 4 to 6 were used to avoid possible variations in cellular function. The SGLT2 was shown to be present in the cells by western blot (Additional file 1: Fig. S1 for SGLT2 protein expression).

### Cell migration assay

Migration of atrial fibroblasts was studied using a wound-healing assay. Briefly, cells were plated in 6-well plates and treated with empagliflozin (1 or 5 μmol/L; MedChemExpress, NJ, USA) or NHE inhibitor (cariporide; 10 μmol/L, Sigma-Aldrich, St. Louis, MO, USA) for 48 h in serum free culture medium for 72 h. Six hours before the end of the treatment, cells were scraped using the tip of a P200 pipette tip. Each area of the gap was assessed using Image J 1.45 s software (National Institute of Health, Bethesda, MD, USA). The net migration area after 6 h was subtracted from that at the time of the initial scratch.

### Cell proliferation assay

Atrial fibroblast proliferation was measured using a commercial MTS kit (Promega, Madison, WI, USA) as previously described [27]. In brief, atrial fibroblasts were seeded onto a 96-well culture dish at a density of 3000 cells/well. After growing to 50% confluence, the cells were incubated with empagliflozin (1 μmol/L, 5 μmol/L) in culture medium for 24 h. Cell growth was analyzed by adding the MTS reagent 4 h before spectrophotometric analysis.

### Western blotting

Western blotting was performed as described previously [28]. Atrial fibroblasts treated with or without empagliflozin (1 or 5 μmol/L), or cariporide (10 μmol/L) for 48 h were lysed in radioimmunoprecipitation assay buffer containing 150 mmol/L NaCl, Nonidet P P40, 50 mmol/L Tris pH 7.4, 0.5% sodium deoxycholate, 0.1% sodium dodecyl sulfate (SDS) and protease inhibitor cocktails (Sigma-Aldrich). The proteins were fractionated using 10% SDS–polyacrylamide gel electrophoresis and transferred onto an equilibrated polyvinylidene difluoride membrane (Amersham Biosciences, Buckinghamshire, UK). Fractionated protein was probed with primary antibodies against α-smooth muscle actin (SMA) (1:1000, monoclonal, clone number: 1A4, Abcam), pro-collagen type IA1(1:500, monoclonal, clone number: 3G3, Santa-Cruz Biotechnology, Santa Cruz, CA, USA), pro-collagen type III(1:1000, monoclonal, clone number: FH7A, Abcam), NHE1 (1:1000, polyclonal, Alomone Labs, Jerusalem, Israel), and phosphorylated PLCγ1 (1:1000, polyclonal, cell signaling, Beverly, MA, USA), followed by incubation with secondary antibodies conjugated with horseradish peroxidase. Bound antibodies were detected using an enhanced chemiluminescence detection system (Millipore, Darmstadt, Germany) and analyzed using AlphaEaseFC software (Alpha Innotech, San Leandro, CA, USA). Glyceraldehyde 3-phosphate dehydrogenase (GAPDH) protein (Sigma-Aldrich) was used as a loading control to confirm equal protein loading and was then normalized to the value of control cells.

### Intracellular pH analysis

Intracellular pH was calculated using a Cell Meter Fluorometric Intracellular pH Assay Kit (AAT Bioquest, Sunnyvale, CA, USA) following the manufacturer’s instructions. In brief, atrial fibroblasts were seeded onto a 96-well culture black plate at a density of 3000 cells/well. After growing to confluence, the cells were incubated with empagliflozin (1 μmol/L) or cariporide (10 μmol/L) for 6 h. The cells were loaded with pH-sensitive cell-permeable fluorescent dye 20,70-biscarboxyethyl-5,6-carboxyfluorescein-acetoxymethyl ester (BCECF-AM) in Hanks’ buffer with 20 mM HEPES and 4.17 mM sodium bicarbonate for 1 h at 37 °C and 5% CO2, in dark. After subsequent washing with phosphate-buffered saline (PBS), fluorescence was measured at excitation/emission wavelengths (Ex/Em) of 505/535 nm and 430/535 nm on a SpectraMax M2 fluorimeter (Molecular Devices, Sunnyvale, CA, USA). The ratio of fluorescence at 505/535 nm and 430/535 nm was converted to a pH unit with Spexyte Intracellular pH Calibration Buffer Kit (AAT Bioquest). Atrial fibroblasts loaded with BCECF-AM were incubated with a range of calibration buffer (pH 4.5 − 8.0) at 37 °C for 10 min with nigericin (10 mmol/L), a proton ionophore that can modulate the intracellular pH with external pH in the presence of 100 − 150 mmol/L K^+^.

### Intracellular Ca^2+^ measurement

The intracellular Ca^2+^ was measured with a ratiometric Ca^2+^ indicator Fura-2 using a fluorescence plate reader as described previously [29]. Atrial fibroblasts were seeded on clear flat-bottom black 96-well culture plates at a density of 5 × 10^3^ cells/well, after incubation overnight, the cells were treated with or without empagliflozin (1 μmol/L) or cariporide (10 μmol/L) for 48 h. Cells were then stained with 5 μmol/L Fura-2 acetoxymethyl ester (Life Technologies, Carlsbad, CA, USA) in a Ca^2+^-free solution with (in mmol/L) KH2PO4 1.2, NaCl 120, MgSO4 1.2, KCl 5.4, HEPES 6, glucose 10 (pH 7.40) for 30 min at 37 °C in a 5% CO2 incubator. Measurements of intracellular Ca^2+^ were performed every 2 s with a fast switching of the excitation wavelengths 340 and 380 nm and a constant emission wavelength of 510 nm using a CLARIOstar PLUS Microplate Reader (BMG Lab Technologies, USA) equipped with two injectors and analyzed with a CLARIOstar MARS software (BMG Lab Technologies). The intracellular Ca^2+^ concentration in each well was expressed as the fluorescence ratio of F340/F380 and the changes of baseline to peak calcium amplitude as well as the area under curve (AUC) of Ca^2+^ tracing were calculated. The decay time of Ca^2+^ entry (T50) was calculated from the peak to the 50% of the decay.

Baseline intracellular Ca^2+^ was recorded for 2 min in Ca^2+^-free buffer, followed by co-treatment with the ER Ca-ATPase inhibitor (thapsigargin, 2.5 μmol/L, Sigma-Aldrich) for ER Ca^2+^ store depletion. After the intracellular Ca^2+^ surge from the thapsigargin-induced ER Ca^2+^ leak returned to a steady state, the extracellular Ca^2+^ concentration was increased to 2 mmol/L to measure Ca^2+^ entry. The change in intracellular Ca^2+^ (∆ F_340_/F_380_) from the steady state of extracellular Ca^2+^-free to the plateau state of thapsigargin cotreatment was defined as the amount of ER Ca^2+^ depletion and the change from the steady state of post-ER Ca^2+^-induced intracellular Ca^2+^ surge to the plateau state under 2 mmol/L Ca^2+^ solution was used to represent Ca^2+^ entry.

### Intracellular inositol trisphosphate (IP3) measurement

Cell lysates of atrial fibroblasts treated with or without empagliflozin (1 μmol/L), or cariporide (10 μmol/L) were assayed for IP3 production using a human IP3 ELISA kit (Amsbio, Abingdon, UK) according to the manufacturer’s instructions. Protein concentrations from the cell lysate of each treatment were used for normalization.

### Effects of empagliflozin on atrial fibrosis in HF

HF induction was conducted as described previously [30]. Male Wistar rats (weighing 300–350 g) were subcutaneously injected with single high dose of isoproterenol (100 mg/kg). 2 weeks after injection, left ventricle fractional shortening (LVFS) of these rats was analyzed by echocardiography. The rats with LVFS < 45% were included in the HF groups [31]. HF rats were then randomly treated with empagliflozin (10 mg/kg/day for 28 days, Jardiance, Boehringer Ingelheim Pharmaceuticals, Ridgefield, CT, USA) or vehicles. After the completion of the 28-day treatment, both treated rats and aged-matched healthy male control rats were euthanized with 5% isoflurane (in oxygen) overdose for histological analysis. All animal protocols conformed to the *Guide for the Care and Use of Laboratory Animals* published by the US National Institutes of Health (NIH Publication No. 85-23, revised 2011) and were approved by the local animal ethics review board (LAC-2021-0223). Animal studies are reported in compliance with the ARRIVE guidelines [32] and with the recommendations made by the British Journal of Pharmacology [33].

Echocardiography was performed before euthanasia. Rats were sedated with 2% isoflurane (in oxygen), placed in the left lateral decubitus position, and scanned using a commercially available echo scanner (Vivid i ultrasound cardiovascular system, GE Healthcare, Haifa, Israel) using a 10S phased array pediatric transducer and a cardiac application with high temporal and spatial resolutions. The transmission frequency was 10 MHz; the depth 2.5 cm; and the frame rate 225 frames/s. LV end-diastolic diameter (LVEDD), LV end-systolic diameter (LVESD), LV posterior wall thickness (LVPW), and heart rate (HR) were measured. The fractional shortening (%) was measured as (LVEDD-LVESD)/LVEDD × 100.

### Serum ß-hydroxybutyrate (ß-OH) analysis

Blood serum was collected before euthanasia, and assayed for ß-OH using a ß-OH fluorometric assay kit (Cayman Chemical Co, Ann Arbor, MI, USA) according to the manufacturer’s instructions.

### Atrial fibrosis analysis

Atrial fibrosis analysis was performed per a previously described method with modification [30]. In brief, left atrial (LA) tissues were fixed in 4% formaldehyde, embedded in paraffin, and stained with Masson’s trichrome staining. Bright-field images of the LA tissues were obtained. LA fibrosis was assessed using collagen volume fraction (the ratio of the total collagen surface area to the total LA surface area). The entire sectioned LA tissues were assessed for collagen deposition blindly with HistoQuest Analysis Software (version 4.0, TissueGnostics, Vienna, Austria).

### Statistical analysis

All quantitative data are expressed as mean ± standard error of the mean. A paired or unpaired t–test for normal distribution, Mann–Whitney rank-sum test for non-normal distribution, and one–way ANOVA or repeated-measures ANOVA or ANOVA on ranks with a post hoc Fisher’s least significant difference (LSD) test were used to compare atrial fibroblasts under different conditions. A *P* < 0.05 was considered statistically significant.

## Results

### Effects of empagliflozin on the profibrotic cellular activities of atrial fibroblasts

**Fig. 1:**
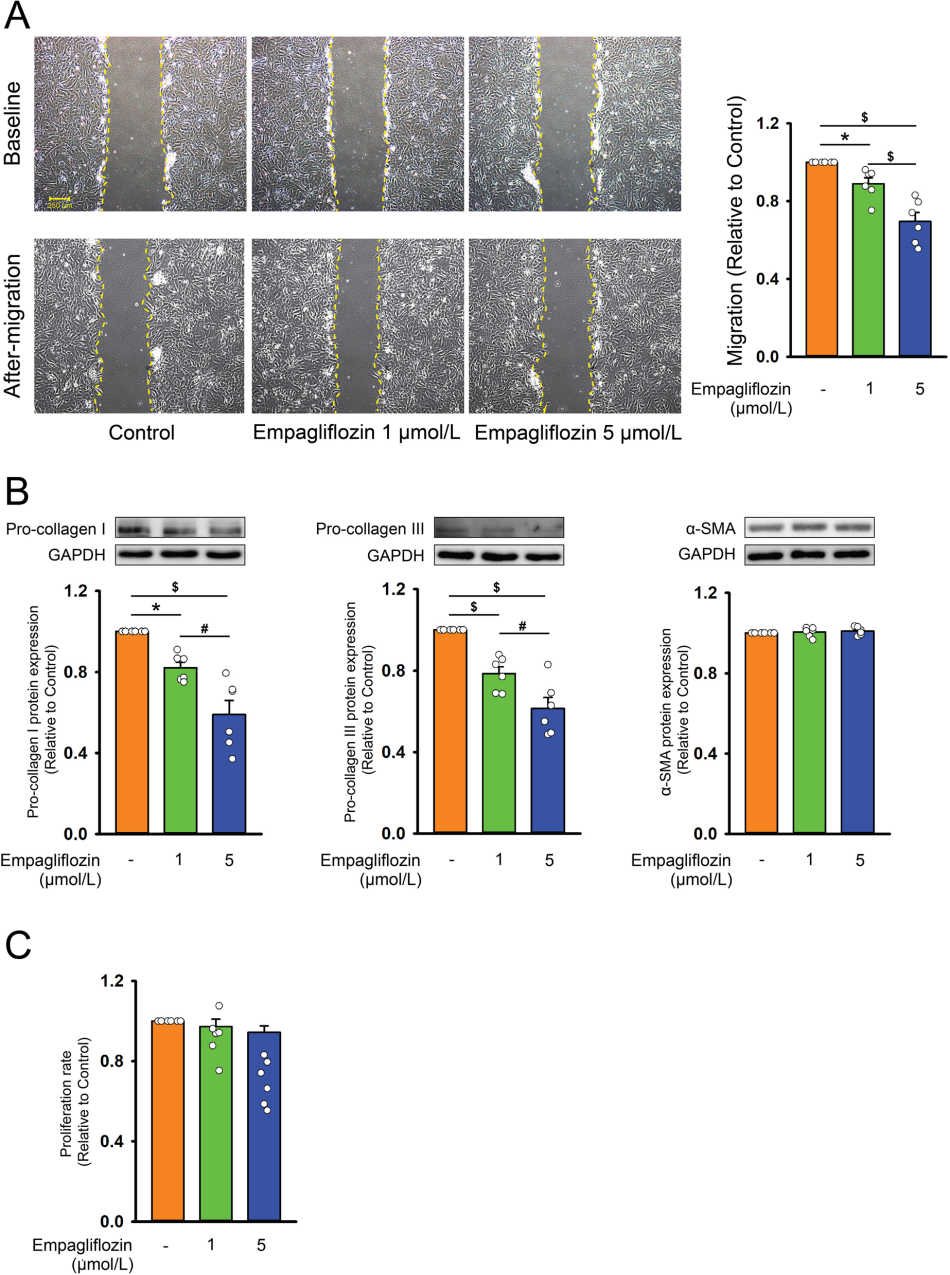
Cell migration, collagen production, myofibroblast differentiation, and proliferation capabilities of atrial fibroblasts treated with empagliflozin. **A** Photographs and averaged data revealed the migration assay results of atrial fibroblasts treated with empagliflozin (1 or 5 μmol/L). The left upper panels displayed the initial scratch (baseline) in different groups. Left lower panels displayed the images 6 h after the scratch was created (after migration) (n = 6 independent experiments, statistic test by one-way repeated measures ANOVA). **B** Photographs and averaged data revealed expression of pro-collagen type I, III, and α-smooth muscle actin (SMA) (n = 6 independent experiments, statistic test by one-way repeated measures ANOVA) in control and empagliflozin (1 or 5 μmol/L)-treated atrial fibroblasts. GAPDH was used as a loading control. **C** Empagliflozin treatment for 24 h had no significant effect on the proliferation rate of atrial fibroblasts (n = 6 independent experiments, statistic test by one-way repeated measures ANOVA). * *p* < 0.05, ^#^ *p* < 0.01, ^$^ *p* < 0.005

Compared with control cells, empagliflozin (1 or 5 μmol/L)-treated atrial fibroblasts exhibited lower migration capability, and lower pro-collagen type I and III protein expression in a dose-dependent manner (Fig. 1). However, control and empagliflozin-treated atrial fibroblasts had similar α-SMA (a myofibroblast differentiation marker) expression, and proliferation rate (Fig. 1).

Figure legend in the result section.

### Ca^2+^ signaling pathway in empagliflozin-treated atrial fibroblasts

**Fig. 2:**
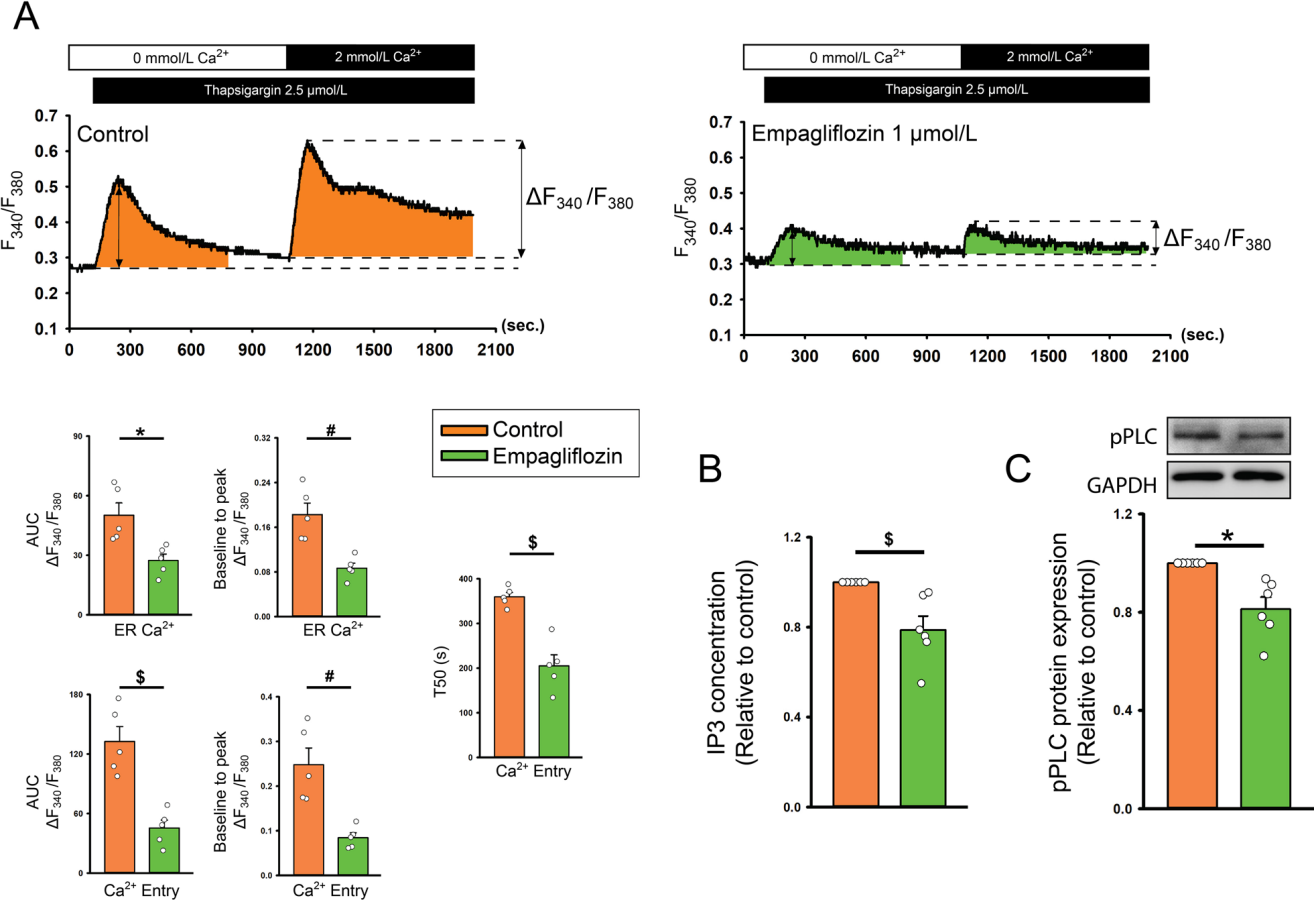
Intracellular Ca^2+^ signaling in empagliflozin-treated atrial fibroblasts. **A** Representative intracellular Ca^2+^ tracing from control (left upper panels), and empagliflozin (1 μmol/L, right upper panels)-treated atrial fibroblasts. Where indicated, thapsigargin was added to the calcium-free buffer to induce ER Ca^2+^ depletion. After the intracellular Ca^2+^ surge induced by thapsigargin (ER calcium) was returned to the steady state, the extracellular Ca^2+^ concentration was then increased to 2 mmol/L to measure Ca^2+^ entry. F_340_/F_380_ was expressed as the relative intracellular Ca^2+^. The left lower panel displays the change (∆ F_340_/F_380_) of Ca^2+^ measured by area under curve of Ca^2+^ tracing (AUC, statistic test by unpaired t-test), the change from baseline to peak calcium amplitude (statistic test by Mann–Whitney Rank Sum Test), and the decay time of Ca^2+^ entry (T50, calculated from the peak to the 50% of the decay or the end of the calcium image recording, statistic test by unpaired t-test) in control (n = 5) and empagliflozin-treated atrial fibroblasts (n = 5). **B** Averaged data of the levels of IP3 in the control cells and fibroblasts treated with empagliflozin (1 μmol/L) for 48 h (*n* = 6 experiments, statistic test by pair t-test). **C** Photographs and averaged data of the expression of phosphorylated phospholipase C (pPLC) in the control cells and fibroblasts treated with empagliflozin (1 μmol/L) for 48 h (n = 6 independent experiments, statistic test by paired t-test). GAPDH was used as a loading control. * *p* < 0.05, ^#^ *p* < 0.01, ^$^ *p* < 0.005

Empagliflozin (1 μmol/L)-treated atrial fibroblasts exhibited lower thapsigargin-induced ER Ca^2+^ leakage, extracellular Ca^2+^ entry, and shorter Ca^2+^ decay during Ca^2+^ entry phase compared with control cells (Fig. 2) Empagliflozin (1 μmol/L) atrial fibroblasts exhibited lower expression of phosphorylated PLC and IP3 (Fig. 2).

### The interaction between empagliflozin with Na^+^-H^+^ exchanger

**Fig. 3:**
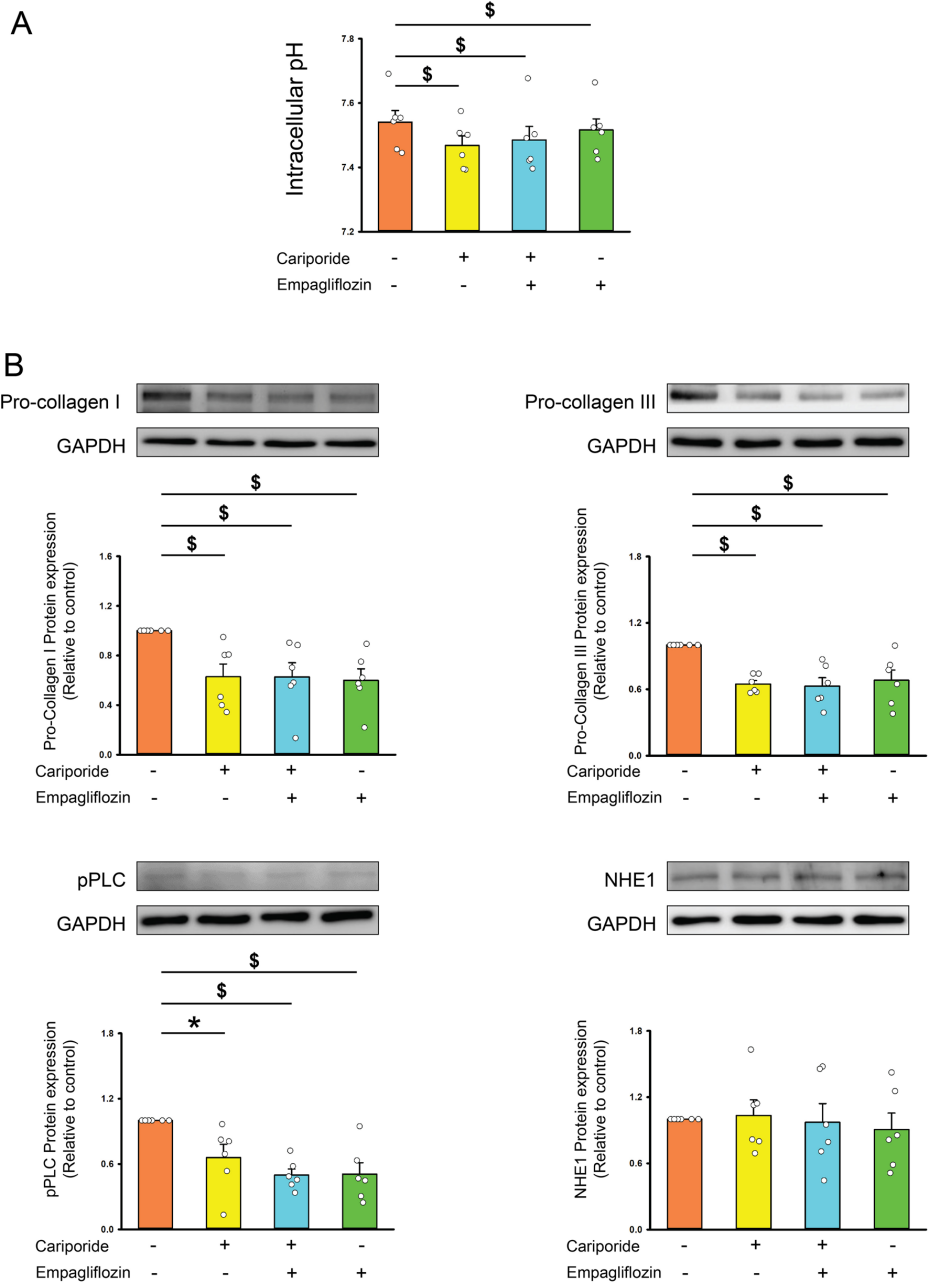
Effects of empagliflozin on Na^+^/H^+^ exchanger (NHE) and downstream signaling. **A** Averaged data of the intracellular pH in the control cells and empagliflozin (1 μmol/L)-treated atrial fibroblasts cotreated with or without cariporide (10 μmol/L) for 48 h (*n* = 6 experiments, statistic test by one-way repeated measures ANOVA). **B** Photographs and averaged data of the expression of pro-collagen type I, type III, phosphorylated phospholipase **C** (pPLC), and NHE1 protein in control cells and empagliflozin (1 μmol/L)-treated atrial fibroblasts cotreated with or without cariporide (10 μmol/L) for 48 h (*n* = 6 experiments, statistic test by one-way repeated measures ANOVA). GAPDH was used as loading control. * *p* < 0.05, ^$^ *p* < 0.005

**Fig. 4:**
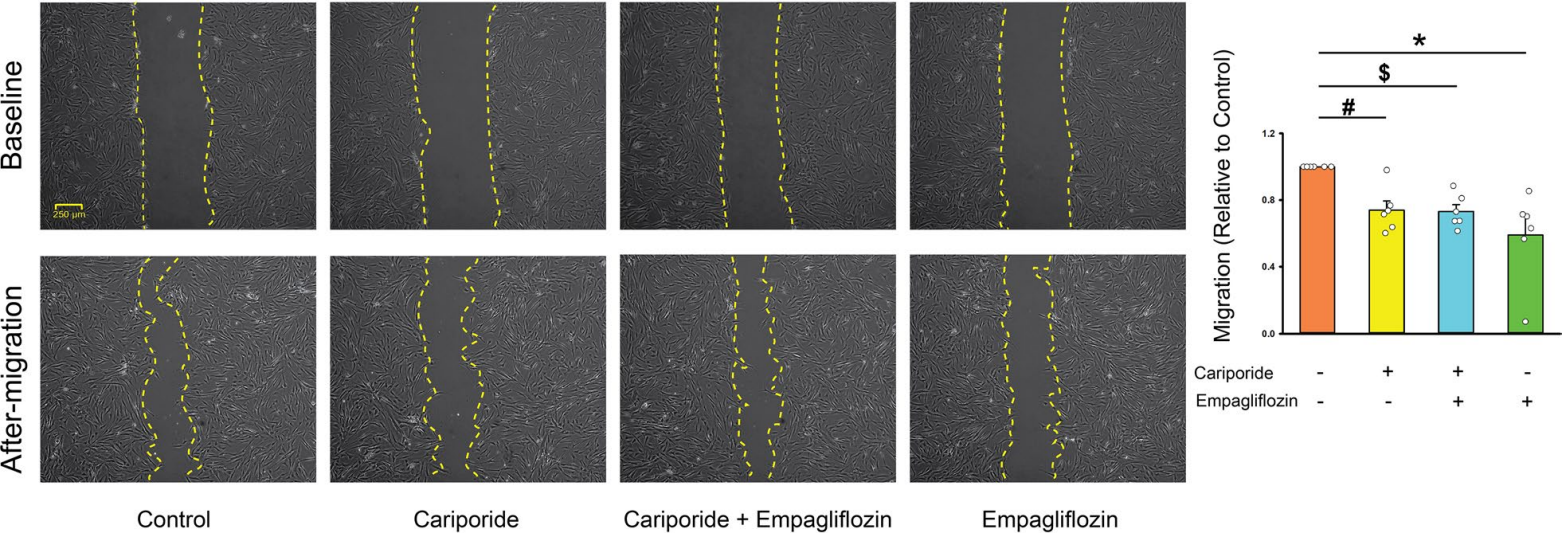
The effects of Na^+^/H^+^ exchanger inhibitor (cariporide) on empagliflozin decreased migration in atrial fibroblasts. Photographs and averaged data present the results of migration assay for empagliflozin (1 μmol/L)-treated atrial fibroblasts treated with or without cariporide (10 μmol/L). Upper panels display the initial scratch (baseline) in different groups. Lower panels display the images 6 h after the scratch was created (after migration) (n = 6 independent experiments, statistic test by one-way repeated measures ANOVA). * *p* < 0.05, ^#^ *p* < 0.01, ^$^ *p* < 0.005

**Fig. 5:**
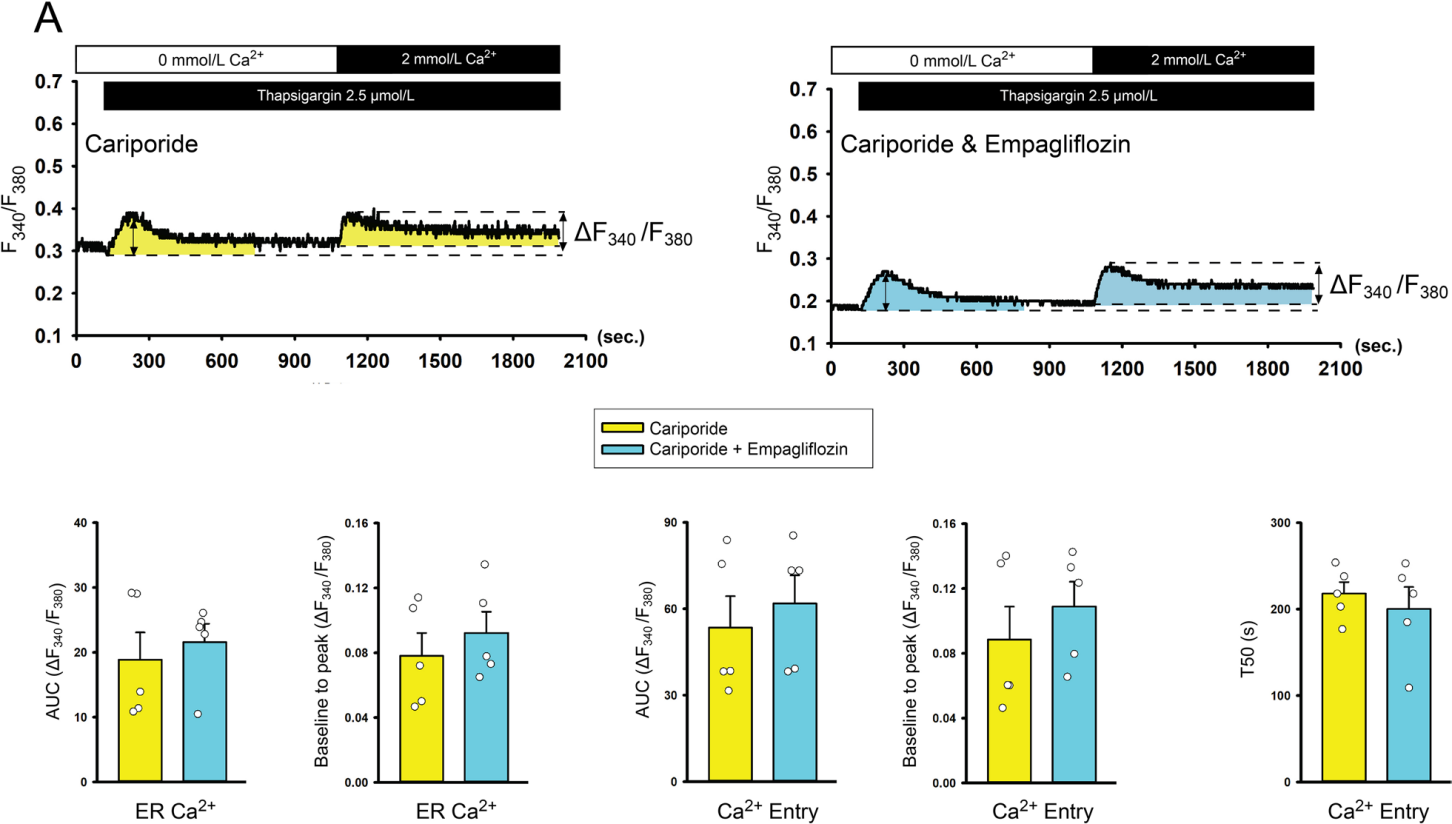
Intracellular Ca^2+^ signaling in empagliflozin or cariporide-treated atrial fibroblasts. Representative intracellular Ca^2+^ tracing from cariporide alone (10 μmol/L, left upper panels), and cariporide (10 μmol/L) mixed with empagliflozin (1 μmol/L, right upper panels). Where indicated, thapsigargin was added to the calcium-free buffer to induce ER Ca^2+^ depletion. After the intracellular Ca^2+^ surge induced by thapsigargin (ER calcium) was returned to the steady state, the extracellular Ca^2+^ concentration was then increased to 2 mmol/L to measure Ca^2+^ entry. F_340_/F_380_ was expressed as the relative intracellular Ca^2+^. The left lower panel displays the change (∆ F_340_/F_380_) of Ca^2+^ measured by area under curve of Ca^2+^ tracing (AUC, statistic test by unpaired t-test), the change from baseline to peak calcium amplitude (statistic test by statistic test by unpaired t-test), and the decay time of Ca^2+^ entry (T50, calculated from the peak to the 50% of the decay or the end of the calcium image recording, statistic test by unpaired t-test) in cariporide alone (n = 5) and cariporide mixed with empagliflozin-treated atrial fibroblasts (n = 5)

Compared to control cells, empagliflozin-treated (1 μmol/L) atrial fibroblasts exhibited lower intracellular pH but had a similar expression of NHE1 protein (Fig. 3). In the presence of cariporide (an NHE inhibitor, 10 μmol/L), the control and empagliflozin-treated atrial fibroblasts exhibited similar levels of intracellular pH, expression of phosphorylated PLC, NHE1, production of type I, type III collagen, migration capability (Figs. 3 and 4), ER Ca^2+^ leakage, extracellular Ca^2+^ entry, and T50 during Ca^2+^ entry phase (Fig. 5), suggesting that empagliflozin decreased the profibrotic activities of atrial fibroblasts by attenuating the PLC/IP3 receptor/ER Ca^2+^ signaling through inhibition of NHE signaling pathway.

### Effects of empagliflozin on heart structure, systolic function, serum ß-OH, and LA fibrosis

**Fig. 6:**
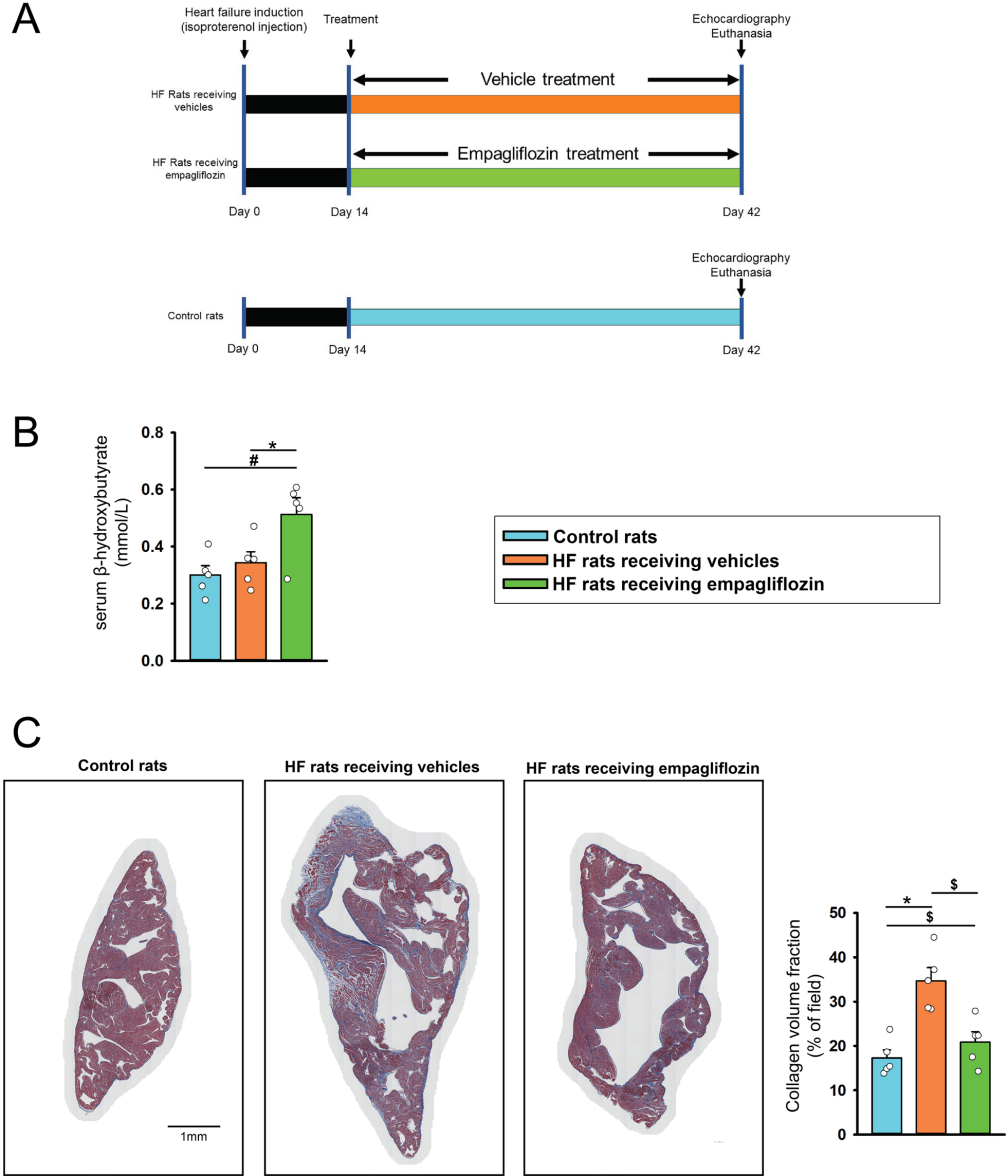
Effects of empagliflozin on rats with isoproterenol-induced heart failure (HF). **A** Schematic summarizing the treatment protocol for Wistar rats with isoproterenol (100 mg/kg, subcutaneous injection)-induced HF receiving vehicles, HF rats receiving empagliflozin (10 mg/kg/day orally for 28 consecutive days), and control rats. **B** averaged data present the results of serum levels of ß-hydroxybutyrate (statistic test by one − way ANOVA) in HF rats receiving vehicles (n = 5), HF rats receiving empagliflozin (n = 5), and control rats (n = 5). **C** Photographs reveal atrial fibrosis (stained with blue color) studied using Masson’s trichrome staining (statistic test by one − way ANOVA) in left atrium (LA) tissues from different groups. Control rats (n = 5) and HF rats receiving empagliflozin (n = 5) exhibited less severe LA fibrosis than vehicle-treated HF rats (n = 5). The fibrosis levels of LA tissues were expressed as the collagen volume fraction, that is, the ratio of the LA total collagen surface area stained blue to the LA total surface area. * *p* < 0.05, ^#^ *p* < 0.01, ^$^ *p* < 0.005

**Fig. 7:**
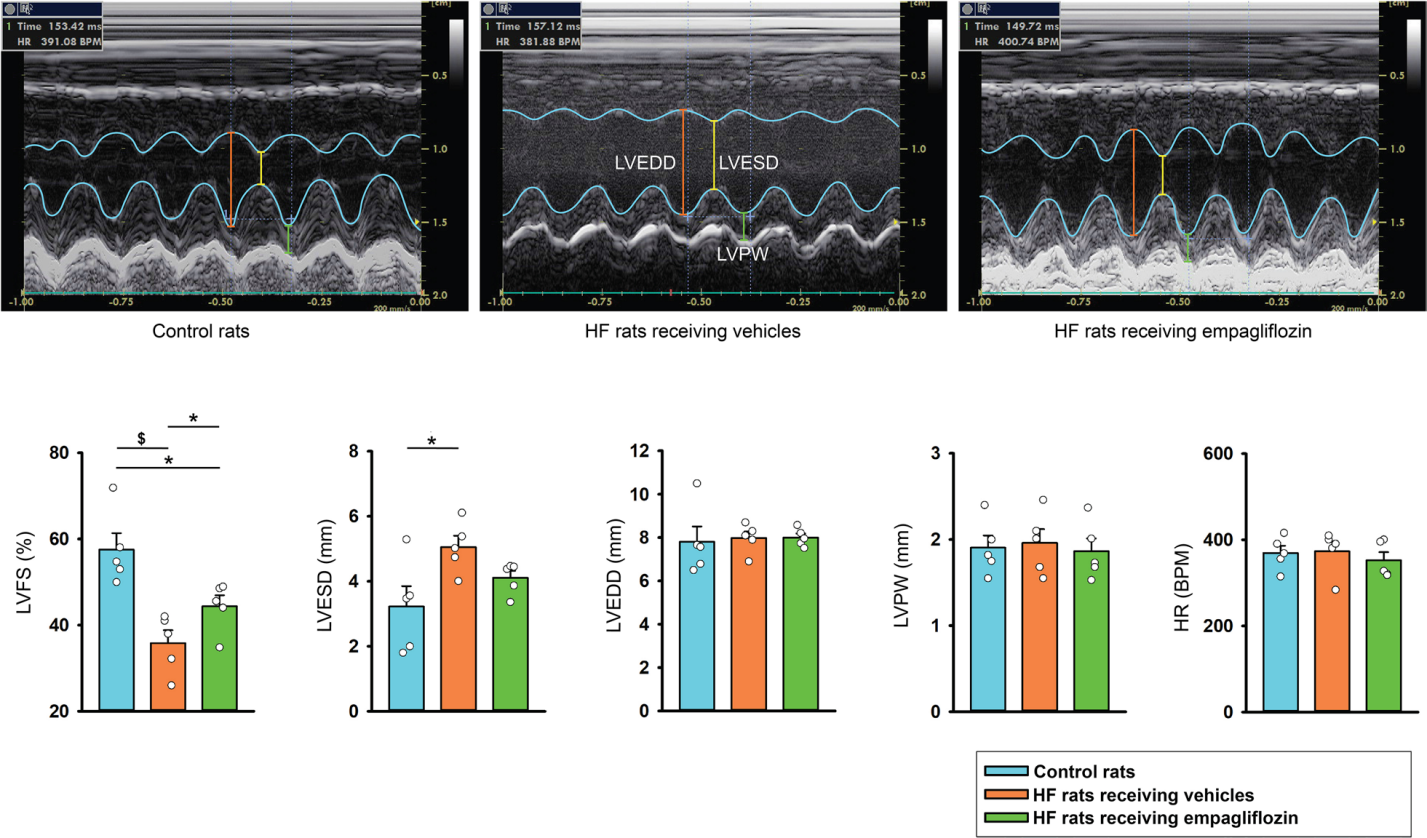
Effects of empagliflozin on heart structure, systolic function, and heart rate of rats with isoproterenol-induced heart failure (HF). Photographs and averaged data present the results of left ventricle fractional shortening (LVFS, statistic test by one − way ANOVA), LV posterior wall (LVPW, statistic test by one − way ANOVA), LV end-diastolic diameter (LVEDD, statistic test by ANOVA on ranks), LV end-systolic diameter (LVESD, statistic test by one − way ANOVA), and heart rate (HR, statistic test by one − way ANOVA) in HF rats receiving vehicles (n = 5), HF rats receiving empagliflozin (n = 5), and control rats (n = 5). * *p* < 0.05, ^$^ *p* < 0.005

As shown in Fig. 6A, we studied the effects of empagliflozin on Heart structure, systolic function, serum ß-OH, and atrial fibrosis in vivo. Echocardiography findings revealed that isoproterenol-treated rats receiving vehicles exhibited lower LV systolic function (LVFS), and LVESD with similar LVPW, LVEDD, and HR than control rats. The isoproterenol-treated rats receiving empagliflozin had higher LV systolic function (LVFS) with similar LVESD, LVEDD, and HR compared to isoproterenol-treated rats receiving vehicles (Fig. 7).

Serum levels of ß-OH revealed that isoproterenol-treated rats receiving empagliflozin have higher serum β-hydroxybutyrate than HF treated with vehicles and healthy control rats before euthanasia (Fig. 6B). Masson’s trichrome staining showed that isoproterenol-treated rats exhibited higher LA fibrosis than control rats and empagliflozin decreased LA fibrosis significantly in isoproterenol-treated rats (Fig. 6C).

## Discussion

Empagliflozin has been demonstrated to improve cardiac fibrosis in various HF experimental models [4], but the underlying mechanisms of the anti-fibrogenic effect have not been fully elucidated. To our knowledge, this is the first study reporting that empagliflozin (1 μmol/L) interrupted Ca^2+^ homeostasis by inhibiting NHE activity in human atrial fibroblasts, thereby reducing their pro-fibrotic cellular activities. Cardiomyocytes with NHE overexpression had a higher intracellular pH. Mice with NHE overexpression exhibited HF and cardiac fibrosis, which can be improved by the NHE1 inhibitor cariporide [34]. In this study, we observed that empagliflozin lowered the intracellular pH of atrial fibroblasts without changing the expression of the NHE1 protein, a predominant isoform in the heart [35]. In addition, cariporide with and without empagliflozin reduced the pro-fibrotic cellular activities of atrial fibroblasts to a similar extent, suggesting that empagliflozin may decrease atrial fibroblast activities by attenuating the activation of NHE signaling.

Elevated intracellular pH activates the PLC/IP3 receptor signaling pathway, thereby inducing ER Ca^2+^ release or Ca^2+^ influx [36]. Pro-fibrotic cytokines induce collagen secretion through the PLC/IP3 receptor signaling pathway, whereas inhibition of the PLC signaling pathway decreases collagen production capability [37]. In the present study, we found that empagliflozin significantly decreased intracellular pH, phosphorylated PLC, and intracellular IP3. Additionally, cariporide with and without empagliflozin reduced the intracellular pH and phosphorylated PLC of atrial fibroblasts to a similar extent. These findings suggest that empagliflozin may attenuate atrial fibrogenesis by inhibiting PLC/IP3 receptor signaling pathway through inhibition of NHE signaling. Similarly, in atrial myocytes, acute exposure to empagliflozin lowered the intracellular pH and attenuated NHE activity. This attenuation was also achieved by incubation with the cariporide [38]. Empagliflozin significantly attenuated NHE flux, thereby reducing cytosolic Na^+^ and Ca^2+^ in ventricular myocytes [39]. In the presence of cariporide, the attenuation effect of empagliflozin was strongly suppressed in atrial fibroblast, suggesting that the effect of NHE inhibition was equivalent to cariporide.

Our previous study found that empagliflozin attenuated sarcoplasmic reticulum (SR) Ca^2+^ content reduction in diabetic myocytes [3]. Besides, empagliflozin can also decrease SR Ca^2+^ leakage [40], suggesting that empagliflozin may decrease ER Ca^2+^ leakage. Inhibition of ER Ca^2+^ leakage interrupts the downstream signaling of pro-fibrotic cytokine [37] The present study found that empagliflozin reduced thapsigargin-induced ER Ca^2+^ release, suggesting that it downregulated the PLC/IP3/ER Ca^2+^ release signaling pathway by decreasing intracellular pH through the inhibition of the NHE, leading to decreased collagen production. Ca^2+^-dependent activation of PLC has been proven in various cells [41, 42]. Perfused ischemic myocardium with a high Ca^2+^ solution increases PLC activity whereas perfusion with a Ca^2+^ channel blocker decreases PLC activity [43]. SGLT2i reduced intracellular Na^+^ by directly docking at the late sodium channel (Nav1.5), inhibiting NHE influx, and reverse mode of NCX thereby decreasing intracellular Ca^2+^ content in cardiomyocytes [39, 44, 45]. In the present study, we found that empagliflozin significantly decreases cytosolic Ca^2+^. Hence, the effect of empagliflozin on lowering PLC activity may also be caused by the decreased cytosolic Ca^2+^ in empagliflozin-treated atrial fibroblasts. The gateways of Ca^2+^ entry include Orai channels, Transient receptor potential (TRP) channels, voltage-operated Ca^2+^ channels, or NCX. Attenuated Orai channel signaling reduces the collagen production capabilities of atrial fibroblasts [46]. Transforming growth factor activate the pro-fibrotic activities of atrial fibroblasts through TRP channels [47]. The emptying of ER Ca^2+^ can activate Orai channels [48, 49]. TRP channels can be activated by PLC signaling [50, 51]. A previous study revealed that SGLT2i decreases high salt-induced vasoconstriction through the inhibition of TRP channels [45]. SGLT2i can also decrease Ca^2+^ overload through the L-type Ca^2+^ channel (a kind of voltage-operated Ca^2+^ channel) in cardiomyocytes [52]. In the present study, we found that empagliflozin decreased ER Ca^2+^ emptying, PLC activity, and extracellular Ca^2+^ entry. Besides, cariporide with and without empagliflozin reduced ER Ca^2+^ leakage, and extracellular Ca^2+^ entry of atrial fibroblasts to a similar extent, suggesting that empagliflozin may decrease suggesting that empagliflozin decreased the cytosolic Ca^2+^ through inhibition of NHE signaling pathway.

Cytoplasmic Ca^2+^ extrusion can be conducted by Ca^2+^ efflux through the plasma membrane ATPases (PMCAs) and forward mode Na^+^/Ca^2+^ exchangers on the cell membrane, or by pumping Ca^2+^ back to ER through SR Ca^2+^-ATPase (SERCA) on the ER membrane. Increasing intracellular pH has been proven to inhibit the ER Ca^2+^ reuptake capability of SERCA [53]. In the present study, we found that empagliflozin shortened the decay time of the Ca^2+^ entry phase. Besides, cariporide with and without empagliflozin shortened the decay time of the Ca^2+^ entry phase of atrial fibroblasts to a similar extent. Hence, empagliflozin might enhance SERCA function and increase the ER Ca^2+^ reuptake thereby shortening the decay time of the Ca^2+^ entry phase of atrial fibroblasts via its effect on decreasing intracellular pH.

The inhibition of NHE by empagliflozin may reduce intracellular Ca^2+^ and decrease the cardiac contraction. However, previous studies have shown that empagliflozin attenuated HF and increased Ca^2+^ content in diabetic cardiomyocytes, which were supposed to arise from its enhancing effects on SERCA expression and L-type calcium currents [3]. Reduction of NHE activity by empagliflozin can reduce oxidative stress, leading to a cardioprotective effect [3]. Moreover, the reduction of fibrogenesis improves HF [54, 55]. Therefore, empagliflozin may improve HF via its anti-fibrosis potential and inotropic effects (enhanced SERCA function and Ca^2+^ content in cardiomyocytes). Isoproterenol activates Ca^2+^/calmodulin-dependent protein kinase II (CaMKII) signaling and induces aberrant Ca^2+^ spark frequency, thereby inducing arrhythmogenesis in failing heart [56, 57]. Empagliflozin modulates various ion channels including Ca^2+^ channels [52], Na^+^ channels [44], and K^+^ channels [58], suggesting that empagliflozin may modulate cardiac electrical activity. Empagliflozin has been shown to reduce ventricular arrhythmia induced by isoproterenol in diabetic heart [59]. Empagliflozin has been proven to decrease Ca^2+^ spark frequency and attenuated CaMKII activities in failing cardiomyocytes [40]. Hence, in our HF models, empagliflozin might alleviate isoproterenol-induced cardiac dysfunction by attenuating SERCA dysfunction or calcium leakage due to phosphorylated ryanodine receptor in HF through reducing oxidative stress or CaMKII activity [3, 40, 60]. Isoproterenol increases HR through beta-adrenergic stimulation in clinical practice. Moreover, long-term treatment of isoproterenol may down-regulate beta-1 adrenergic receptors, leading to a decrease or loss of efficacy of beta-adrenergic receptor agonists [61]. Isoproterenol has been widely used for HF animal model induction. However, the significant increase in HR post HF induction with isoproterenol is not consistent between different studies [62, 63]. In the present study, we found that there is no significant difference between healthy control rats and HF rats, which is possibly attributed to the frequency of isoproterenol injection (once only) and the timing of heart rate measuring (6 weeks after isoproterenol injection).

DM patients treated with SGLT2i exhibited higher levels of ketone (β-OHB) [64, 65]. SGLT2i activates lipolysis and decreases insulin levels, thereby driving ketone production in the liver [66]. β-OHB can also improve the cardiac output and systolic function of patients with HF [67]. In the present study, we found that LV systolic function and serum level of β-OHB are higher in HF rats treated with empagliflozin than in HF rats treated with vehicles, suggesting that β-OHB might contribute to the cardioprotective effects of empagliflozin.

More severe atrial fibrosis is associated with a higher incidence of atrial fibrillation [68]. Empagliflozin decreased ventricular fibrosis in diabetic rats [69]. In the present study, empagliflozin reduced atrial fibrosis in isoproterenol-treated rats, we found that empagliflozin reduced atrial fibrosis. To correlate with the clinical settings, we treated atrial fibroblasts with 1 μmol/L empagliflozin (a concentration similar to the maximal plasma empagliflozin concentration in patients with type 2 diabetes after intake of multiple oral doses [70, 71]). This finding indicated the clinical relevance of the anti-fibrogenic effect of empagliflozin in human atrial fibroblasts. Accordingly, SGLT-2i may be a potential therapeutic strategy.

## Study limitations

There were a few limitations in this study. First, SGLT2i improved end-systolic and end-diastolic pressure–volume relationships in diabetic animal models according to pressure–volume loop experiments [72]. However, this study did not conduct this experiment. Hence, it is not clear about the effects of SGLT2i on activation or relaxation behavior of HF myocardium. In addition, compared to control cells, atrial fibroblasts exhibited lower intracellular pH under 6 h of empagliflozin treatment through NHE signaling. Nevertheless, it remains unclear how long empagliflozin takes to work for the intracellular pH modification in atrial fibroblasts. Moreover, this study measured chamber size and heart function 6 weeks after isoproterenol treatment. The similar thickness between control and HF rats might be attributed to the net results of the loss of myocardium and compensatory ventricular myocyte hypertrophy. A previous study revealed that young adult wistar HF rats induced by high dose (170 mg/kg/d) of isoproterenol had a similar thickness of LV posterior wall 4 weeks and 8 weeks after HF induction [73]. Similarly, HF induced by 85 or 170 mg/kg/d of isoproterenol in wistar rats had increasing LV mass only at 16 weeks, but not 2 or 6 weeks post HF induction [74]. These findings suggest that ventricular hypertrophy in isoproterenol-induced HF may be dose and duration dependent. Thus, longer duration after isoproterenol treatment may have a different impact on LV posterior wall thickness. Finally, to mimic the clinical scenario, we did not measure the effect of empagliflozin on control rat hearts, and the effects of empagliflozin on healthy animals are not elucidated in this study. However, studies revealed that empagliflozin may not affect cardiac fibrosis, ventricular wall thickness, and ejection fraction of healthy rats [75, 76].

**Fig. 8:**
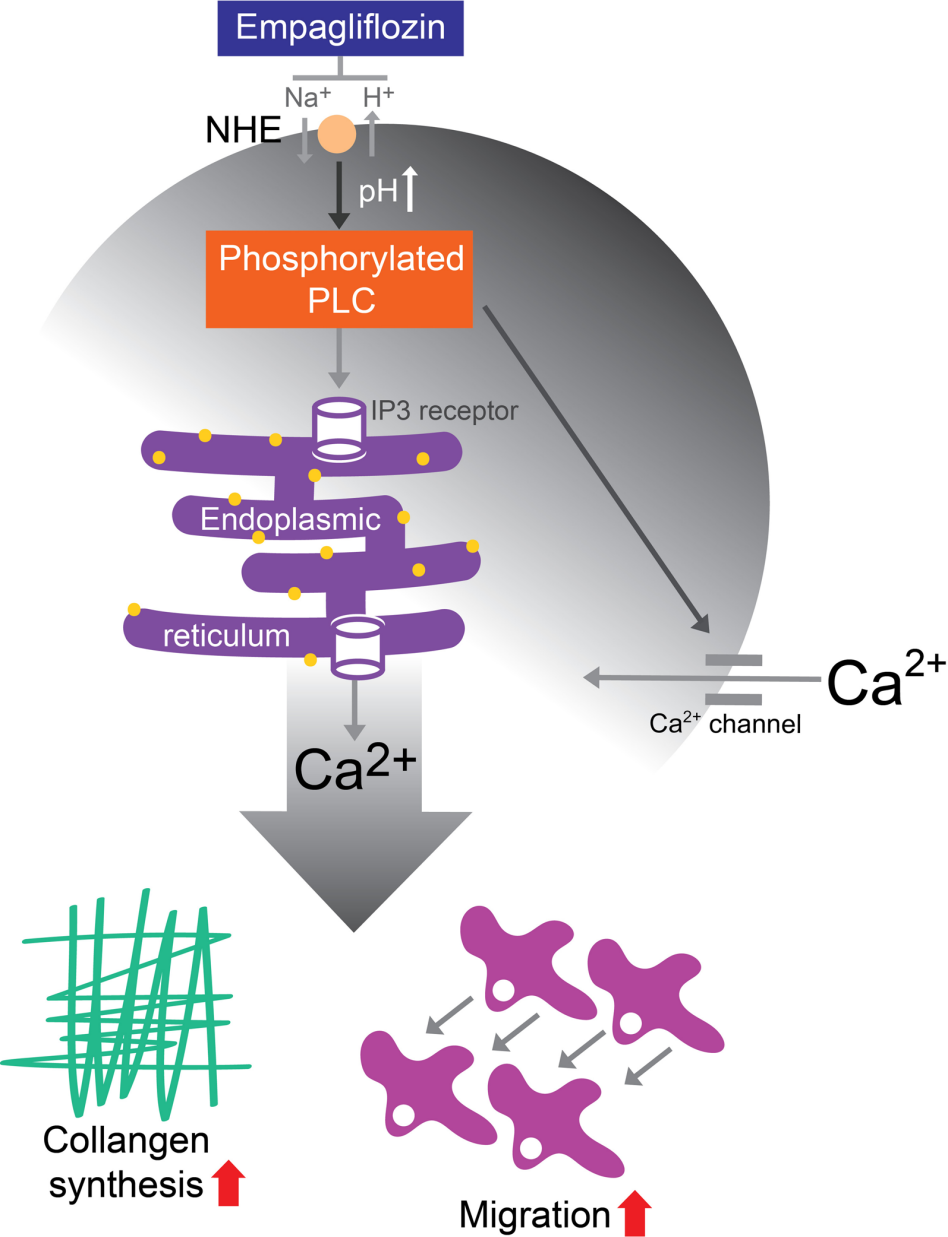
The proposed molecular mechanism underlying the anti-fibrotic effects of empagliflozin on atrial fibroblasts. By inhibiting the Na^+^-H^+^ exchanger (NHE), empagliflozin decreases the expression of phosphorylated phospholipase C (PLC) and inositol trisphosphate (IP3) production thereby reducing ER Ca^2+^ release, extracellular Ca^2+^ entry and decreasing profibrotic cellular activities of atrial fibroblasts

In conclusion, as summarized in Fig. 8, by inhibiting NHE, empagliflozin decreases the expression of phosphorylated PLC and IP3 production, thereby reducing ER Ca^2+^ release, extracellular Ca^2+^ entry and the profibrotic activities of atrial fibroblasts.

## Supplementary Information

**Additional file 1 : Fig. S1. SGLT2 protein is expressed in human atrial fibroblasts.**

## Data Availability

The data that support the findings of this study are available from the corresponding author upon reasonable request.

## References

[1] Packer M, Anker SD, Butler J, Filippatos G, Pocock SJ, Carson P, Januzzi J, Verma S, Tsutsui H, Brueckmann M (2020). Cardiovascular and renal outcomes with empagliflozin in heart failure. N Engl J Med.

[2] Zinman B, Wanner C, Lachin JM, Fitchett D, Bluhmki E, Hantel S, Mattheus M, Devins T, Johansen OE, Woerle HJ (2015). Empagliflozin, cardiovascular outcomes, and mortality in type 2 diabetes. N Engl J Med.

[3] Lee TI, Chen YC, Lin YK, Chung CC, Lu YY, Kao YH, Chen YJ (2019). Empagliflozin attenuates myocardial Sodium and calcium dysregulation and reverses cardiac remodeling in streptozotocin-induced diabetic rats. Int J Mol Sci.

[4] Santos-Gallego CG, Requena-Ibanez JA, San Antonio R, Garcia-Ropero A, Ishikawa K, Watanabe S, Picatoste B, Vargas-Delgado AP, Flores-Umanzor EJ, Sanz J (2021). Empagliflozin ameliorates diastolic dysfunction and left ventricular fibrosis/stiffness in nondiabetic heart failure: a multimodality study. JACC Cardiovasc Imaging.

[5] Kostin S, Klein G, Szalay Z, Hein S, Bauer EP, Schaper J (2002). Structural correlate of atrial fibrillation in human patients. Cardiovasc Res.

[6] Dosdall DJ, Ranjan R, Higuchi K, Kholmovski E, Angel N, Li L, Macleod R, Norlund L, Olsen A, Davies CJ (2013). Chronic atrial fibrillation causes left ventricular dysfunction in dogs but not goats: experience with dogs, goats, and pigs. Am J Physiol Heart Circ Physiol.

[7] Omar M, Jensen J, Frederiksen PH, Kistorp C, Videbæk L, Poulsen MK, Möller S, Ali M, Gustafsson F, Køber L (2020). Effect of empagliflozin on effect of empagliflozin on hemodynamics in patients with heart failure and reduced ejection fraction. J Am Coll Cardiol.

[8] Núñez J, Palau P, Domínguez E, Mollar A, Núñez E, Ramón JM, Miñana G, Santas E, Fácila L, Górriz JL (2018). Early effects of empagliflozin on exercise tolerance in patients with heart failure: a pilot study. Clin Cardiol.

[9] Pabel S, Wagner S, Bollenberg H, Bengel P, Kovács Á, Schach C, Tirilomis P, Mustroph J, Renner A, Gummert J (2018). Empagliflozin directly improves diastolic function in human heart failure. Eur J Heart Fail.

[10] Clauss S, Schüttler D, Bleyer C, Vlcek J, Shakarami M, Tomsits P, Schneider S, Maderspacher F, Chataut K, Trebo A (2020). Characterization of a porcine model of atrial arrhythmogenicity in the context of ischaemic heart failure. PLoS ONE.

[11] Malmo V, Kelly A, Garten KS, Stolen T, Rolim NPL, Wisloff U, Smith G, Loennechen JP (2018). Aerobic interval training prevents age-dependent vulnerability to atrial fibrillation in rodents. Front Physiol.

[12] Thomas L, Marwick TH, Popescu BA, Donal E, Badano LP (2019). Left atrial structure and function, and left ventricular diastolic dysfunction: JACC state-of-the-Art review. J Am Coll Cardiol.

[13] Ikeda K, Nakajima T, Yamamoto Y, Takano N, Tanaka T, Kikuchi H, Oguri G, Morita T, Nakamura F, Komuro I (2013). Roles of transient receptor potential canonical (TRPC) channels and reverse-mode Na^+^/Ca^2+^ exchanger on cell proliferation in human cardiac fibroblasts: effects of transforming growth factor β1. Cell Calcium.

[14] Brilla CG, Scheer C, Rupp H (1998). Angiotensin II and intracellular calcium of adult cardiac fibroblasts. J Mol Cell Cardiol.

[15] Yang S, Huang XY (2005). Ca^2+^ influx through L-type Ca2+ channels controls the trailing tail contraction in growth factor-induced fibroblast cell migration. J Biol Chem.

[16] Zhang B, Jiang J, Yue Z, Liu S, Ma Y, Yu N, Gao Y, Sun S, Chen S, Liu P (2016). Store-operated Ca^2+^ entry (SOCE) contributes to angiotensin II-induced cardiac fibrosis in cardiac fibroblasts. J Pharmacol Sci.

[17] Uthman L, Baartscheer A, Bleijlevens B, Schumacher CA, Fiolet JWT, Koeman A, Jancev M, Hollmann MW, Weber NC, Coronel R (2018). Class effects of SGLT2 inhibitors in mouse cardiomyocytes and hearts: inhibition of Na(+)/H(+) exchanger, lowering of cytosolic Na(+) and vasodilation. Diabetologia.

[18] Li S, Hao B, Lu Y, Yu P, Lee H-C, Yue J (2012). Intracellular alkalinization induces cytosolic Ca^2+^ increases by inhibiting sarco/endoplasmic reticulum Ca^2+^-ATPase (SERCA). PLoS ONE.

[19] Eto W, Hirano K, Hirano M, Nishimura J, Kanaide H (2003). Intracellular alkalinization induces Ca^2+^ influx via non-voltage-operated Ca^2+^ channels in rat aortic smooth muscle cells. Cell Calcium.

[20] Pérez NG, Alvarez BV, Camilión de Hurtado MC, Cingolani HE (1995). pHi regulation in myocardium of the spontaneously hypertensive rat compensated enhanced activity of the Na(+)-H+ exchanger. Circ Res.

[21] Yokoyama H, Gunasegaram S, Harding SE, Avkiran M (2000). Sarcolemmal Na^+^/H^+^ exchanger activity and expression in human ventricular myocardium. J Am Coll Cardiol.

[22] Aker S, Snabaitis AK, Konietzka I, van de Sand A, Böngler K, Avkiran M, Heusch G, Schulz R (2004). Inhibition of the Na^+^/H^+^ exchanger attenuates the deterioration of ventricular function during pacing-induced heart failure in rabbits. Cardiovasc Res.

[23] Engelhardt S, Hein L, Keller U, Klämbt K, Lohse MJ (2002). Inhibition of Na^+^-H^+^ exchange prevents hypertrophy, fibrosis, and heart failure in β1-adrenergic receptor transgenic mice. Circ Res.

[24] Ennis Irene L, Escudero Eduardo M, Console Gloria M, Camihort G, Dumm César G, Seidler Randolph W, de Hurtado C, María C, Cingolani HE (2003). Regression of isoproterenol-induced cardiac hypertrophy by Na^+^/H^+^ exchanger inhibition. Hypertension.

[25] Denker SP, Barber DL (2002). Cell migration requires both ion translocation and cytoskeletal anchoring by the Na-H exchanger NHE1. J Cell Biol.

[26] Ye Y, Jia X, Bajaj M, Birnbaum Y (2018). Dapagliflozin attenuates Na(+)/H(+) exchanger-1 in cardiofibroblasts via AMPK activation. Cardiovasc Drugs Ther.

[27] Chung CC, Hsu RC, Kao YH, Liou JP, Lu YY, Chen YJ (2014). Androgen attenuates cardiac fibroblasts activations through modulations of transforming growth factor-β and angiotensin II signaling. Int J Cardiol.

[28] Chung CC, Kao YH, Yao CJ, Lin YK, Chen YJ (2017). A comparison of left and right atrial fibroblasts reveals different collagen production activity and stress-induced mitogen-activated protein kinase signalling in rats. Acta Physiol.

[29] Martínez M, Martínez NA, Silva WI (2017). Measurement of the intracellular calcium concentration with fura-2 AM using a fluorescence plate reader. Bio Protoc.

[30] Chung CC, Lin YK, Chen YC, Kao YH, Yeh YH, Chen YJ (2018). Factor Xa inhibition by rivaroxaban regulates fibrogenesis in human atrial fibroblasts with modulation of nitric oxide synthesis and calcium homeostasis. J Mol Cell Cardiol.

[31] Watson LE, Sheth M, Denyer RF, Dostal DE (2004). Baseline echocardiographic values for adult male rats. J Am Soc Echocardiogr.

[32] Percie du Sert N, Hurst V, Ahluwalia A, Alam S, Avey MT, Baker M, Browne WJ, Clark A, Cuthill IC, Dirnagl U (2020). The ARRIVE guidelines 2 0: updated guidelines for reporting animal research. Br J Pharmacol.

[33] Lilley E, Stanford SC, Kendall DE, Alexander SPH, Cirino G, Docherty JR, George CH, Insel PA, Izzo AA, Ji Y (2020). ARRIVE 2 0 and the british journal of pharmacology: updated guidance for 2020. Br J Pharmacol.

[34] Nakamura Tomoe Y, Iwata Y, Arai Y, Komamura K, Wakabayashi S (2008). Activation of Na^+^/H^+^ exchanger 1 is sufficient to generate Ca^2+^ signals that induce cardiac hypertrophy and heart failure. Circ Res.

[35] Padan E, Landau M (2016). Sodium-proton (Na(+)/H(+)) antiporters: properties and roles in health and disease. Met Ions Life Sci.

[36] Minelli A, Lyons S, Nolte C, Verkhratsky A, Kettenmann H (2000). Ammonium triggers calcium elevation in cultured mouse microglial cells by initiating Ca^2+^ release from thapsigargin-sensitive intracellular stores. Pflugers Arch.

[37] Mukherjee S, Duan F, Kolb MRJ, Janssen LJ (2013). Platelet derived growth factor-evoked Ca^2+^ wave and matrix gene expression through phospholipase C in human pulmonary fibroblast. Int J Biochem Cell Biol.

[38] Trum M, Riechel J, Lebek S, Pabel S, Sossalla ST, Hirt S, Arzt M, Maier LS, Wagner S (2020). Empagliflozin inhibits Na(+) /H(+) exchanger activity in human atrial cardiomyocytes. ESC Heart Fail.

[39] Baartscheer A, Schumacher CA, Wüst RC, Fiolet JW, Stienen GJ, Coronel R, Zuurbier CJ (2017). Empagliflozin decreases myocardial cytoplasmic Na(+) through inhibition of the cardiac Na(+)/H(+) exchanger in rats and rabbits. Diabetologia.

[40] Mustroph J, Wagemann O, Lücht CM, Trum M, Hammer KP, Sag CM, Lebek S, Tarnowski D, Reinders J, Perbellini F (2018). Empagliflozin reduces Ca/calmodulin-dependent kinase II activity in isolated ventricular cardiomyocytes. ESC Heart Fail.

[41] Ryan MJ, Gross KW, Hajduczok G (2000). Calcium-dependent activation of phospholipase C by mechanical distension in renin-expressing As4.1 cells. Am J Physiol Endocrinol Metab.

[42] Gusovsky F, Lueders JE, Kohn EC, Felder CC (1993). Muscarinic receptor-mediated tyrosine phosphorylation of phospholipase C-gamma an alternative mechanism for cholinergic-induced phosphoinositide breakdown. J Biol Chem.

[43] Asemu G, Dhalla NS, Tappia PS (2004). Inhibition of PLC improves postischemic recovery in isolated rat heart. Am J Physiol Heart Circ Physiol.

[44] Philippaert K, Kalyaanamoorthy S, Fatehi M, Long W, Soni S, Byrne NJ, Barr A, Singh J, Wong J, Palechuk T (2021). Cardiac late sodium channel current is a molecular target for the sodium/glucose cotransporter 2 inhibitor empagliflozin. Circulation.

[45] Zhao Y, Li L, Lu Z, Hu Y, Zhang H, Sun F, Li Q, He C, Shu W, Wang L (2022). Sodium-glucose cotransporter 2 inhibitor canagliflozin antagonizes salt-sensitive hypertension through modifying transient receptor potential channels 3 mediated vascular calcium handling. J Am Heart Assoc.

[46] Chen PH, Chung CC, Lin YF, Kao YH, Chen YJ (2021). Lithium reduces migration and collagen synthesis activity in human cardiac fibroblasts by inhibiting store-operated Ca(2+) entry. Int J Mol Sci.

[47] Du J, Xie J, Zhang Z, Tsujikawa H, Fusco D, Silverman D, Liang B, Yue L (2010). TRPM7-mediated Ca(2+) signals confer fibrogenesis in human atrial fibrillation. Circ Res.

[48] Stathopulos PB, Zheng L, Li GY, Plevin MJ, Ikura M (2008). Structural and mechanistic insights into stim1-mediated initiation of store-operated calcium entry. Cell.

[49] Muik M, Schindl R, Fahrner M, Romanin C (2012). Ca(2+) release-activated Ca(2+) (CRAC) current, structure, and function. Cell Mol Life Sci CMLS.

[50] Wedel B, Boyles RR, Putney JW, Bird GS (2007). Role of the store-operated calcium entry proteins stim1 and orai1 in muscarinic cholinergic receptor-stimulated calcium oscillations in human embryonic kidney cells. The J Physiol.

[51] Hofmann T, Obukhov AG, Schaefer M, Harteneck C, Gudermann T, Schultz G (1999). Direct activation of human TRPC6 and TRPC3 channels by diacylglycerol. Nature.

[52] Jhuo SJ, Liu IH, Tsai WC, Chou TW, Lin YH, Wu BN, Lee KT, Lai WT (2020). Effects of secretome from fat tissues on ion currents of cardiomyocyte modulated by sodium-glucose transporter 2 inhibitor. Molecules.

[53] Li S, Hao B, Lu Y, Yu P, Lee HC, Yue J (2012). Intracellular alkalinization induces cytosolic Ca2+ increases by inhibiting sarco/endoplasmic reticulum Ca^2+^-ATPase (SERCA). PLoS ONE.

[54] Kao YH, Liou JP, Chung CC, Lien GS, Kuo CC, Chen SA, Chen YJ (2013). Histone deacetylase inhibition improved cardiac functions with direct antifibrotic activity in heart failure. Int J Cardiol.

[55] Liang H, Pan Z, Zhao X, Liu L, Sun J, Su X, Xu C, Zhou Y, Zhao D, Xu B (2018). LncRNA PFL contributes to cardiac fibrosis by acting as a competing endogenous RNA of let-7d. Theranostics.

[56] Murakami W, Kobayashi S, Susa T, Nanno T, Ishiguchi H, Myoren T, Nishimura S, Kato T, Hino A, Oda T (2016). Recombinant atrial natriuretic peptide prevents aberrant Ca2+ leakage through the ryanodine receptor by suppressing mitochondrial reactive oxygen species production induced by isoproterenol in failing cardiomyocytes. PLoS ONE.

[57] Park SW, Nhieu J, Lin YW, Wei LN (2019). All-trans retinoic acid attenuates isoproterenol-induced cardiac dysfunction through Crabp1 to dampen CaMKII activation. Eur J Pharmacol.

[58] Karpushev AV, Mikhailova VB, Klimenko ES, Kulikov AN, Ivkin DY, Kaschina E, Okovityi SV (2022). SGLT2 inhibitor empagliflozin modulates ion channels in adult zebrafish heart. Int J Mol Sci.

[59] Kadosaka T, Watanabe M, Natsui H, Koizumi T, Koya T, Nakao M, Hagiwara H, Kamada R, Temma T, Anzai T (2022). Empagliflozin attenuates arrhythmogenesis via inhibition of O-GlcNAcylation in diastolic phase of diabetic cardiomyopathy. Eur Heart J.

[60] Braun JL, Hamstra SI, Messner HN, Fajardo VA (2019). SERCA2a tyrosine nitration coincides with impairments in maximal SERCA activity in left ventricles from tafazzin-deficient mice. Physiol Rep.

[61] Yin Q, Yang C, Wu J, Lu H, Zheng X, Zhang Y, Lv Z, Zheng X, Li Z (2016). Downregulation of β-adrenoceptors in isoproterenol-induced cardiac remodeling through HuR. PLoS ONE.

[62] Elasoru SE, Rhana P, de Oliveira BT, Naves de Souza DL, Menezes-Filho JER, Souza DS, Loes Moreira MV, Gomes Campos MT, Adedosu OT, Roman-Campos D (2021). Andrographolide protects against isoproterenol-induced myocardial infarction in rats through inhibition of L-type Ca(2+) and increase of cardiac transient outward K(+) currents. Eur J Pharmacol.

[63] Parveen A, Babbar R, Agarwal S, Kotwani A, Fahim M (2012). Terminalia arjuna enhances baroreflex sensitivity and myocardial function in isoproterenol-induced chronic heart failure rats. J Cardiovasc Pharmacol Ther.

[64] Ferrannini E, Baldi S, Frascerra S, Astiarraga B, Heise T, Bizzotto R, Mari A, Pieber TR, Muscelli E (2016). Shift to fatty substrate utilization in response to sodium-glucose cotransporter 2 inhibition in subjects without diabetes and patients with type 2 diabetes. Diabetes.

[65] Shimada A, Hanafusa T, Yasui A, Lee G, Taneda Y, Sarashina A, Shiki K, George J, Soleymanlou N, Marquard J (2018). Empagliflozin as adjunct to insulin in Japanese participants with type 1 diabetes: results of a 4-week, double-blind, randomized, placebo-controlled phase 2 trial. Diabetes Obes Metab.

[66] Bonner C, Kerr-Conte J, Gmyr V, Queniat G, Moerman E, Thévenet J, Beaucamps C, Delalleau N, Popescu I, Malaisse WJ (2015). Inhibition of the glucose transporter SGLT2 with dapagliflozin in pancreatic alpha cells triggers glucagon secretion. Nat Med.

[67] Nielsen R, Møller N, Gormsen LC, Tolbod LP, Hansson NH, Sorensen J, Harms HJ, Frøkiær J, Eiskjaer H, Jespersen NR (2019). Cardiovascular effects of treatment with the ketone body 3-hydroxybutyrate in chronic heart failure patients. Circulation.

[68] Oakes RS, Badger TJ, Kholmovski EG, Akoum N, Burgon NS, Fish EN, Blauer JJE, Rao SN, DiBella EVR, Segerson NM (2009). Detection and quantification of left atrial structural remodeling with delayed-enhancement magnetic resonance imaging in patients with atrial fibrillation. Circulation.

[69] Trang NN, Chung CC, Lee TW, Cheng WL, Kao YH, Huang SY, Lee TI, Chen YJ (2021). Empagliflozin and liraglutide differentially modulate cardiac metabolism in diabetic cardiomyopathy in rats. Int J Mol Sci.

[70] Heise T, Seewaldt-Becker E, Macha S, Hantel S, Pinnetti S, Seman L, Woerle HJ (2013). Safety, tolerability, pharmacokinetics and pharmacodynamics following 4 weeks’ treatment with empagliflozin once daily in patients with type 2 diabetes. Diabetes Obes Metab.

[71] Heise T, Seman L, Macha S, Jones P, Marquart A, Pinnetti S, Woerle HJ, Dugi K (2013). Safety, tolerability, pharmacokinetics, and pharmacodynamics of multiple rising doses of empagliflozin in patients with type 2 diabetes mellitus. Diabetes Ther.

[72] Hammoudi N, Jeong D, Singh R, Farhat A, Komajda M, Mayoux E, Hajjar R, Lebeche D (2017). Empagliflozin improves left ventricular diastolic dysfunction in a genetic model of type 2 diabetes. Cardiovasc Drugs Ther.

[73] Razavi Tousi SM, Faghihi M, Nobakht M, Molazem M, Kalantari E, Darbandi Azar A, Aboutaleb N (2016). Improvement of heart failure by human amniotic mesenchymal stromal cell transplantation in rats. J Tehran Heart Cent.

[74] Teerlink JR, Pfeffer JM, Pfeffer MA (1994). Progressive ventricular remodeling in response to diffuse isoproterenol-induced myocardial necrosis in rats. Circ Res.

[75] Li X, Lu Q, Qiu Y, do Carmo JM, Wang Z, da Silva AA, Mouton A, Omoto ACM, Hall ME, Li J (2021). direct cardiac actions of the sodium glucose co transporter 2 inhibitor empagliflozin improve myocardial oxidative phosphorylation and attenuate pressure overload heart failure. J Am Heart Assoc.

[76] Connelly KA, Zhang Y, Desjardins JF, Nghiem L, Visram A, Batchu SN, Yerra VG, Kabir G, Thai K, Advani A (2020). Load-independent effects of empagliflozin contribute to improved cardiac function in experimental heart failure with reduced ejection fraction. Cardiovasc Diabetol.

