# Supplementary material for: Empagliflozin suppressed cardiac fibrogenesis through sodium-hydrogen exchanger inhibition and modulation of the calcium homeostasis
Source: Cardiovasc Diabetol. 2023 Feb 6;22:27. doi: 10.1186/s12933-023-01756-0 (PMC9903522; doi:10.1186/s12933-023-01756-0)
Supplement: Supplementary file 1 — Additional file 1 : Fig. S1. SGLT2 protein is expressed in human atrial fibroblasts. [file 12933_2023_1756_MOESM1_ESM.pdf]

Additional Fig. S1

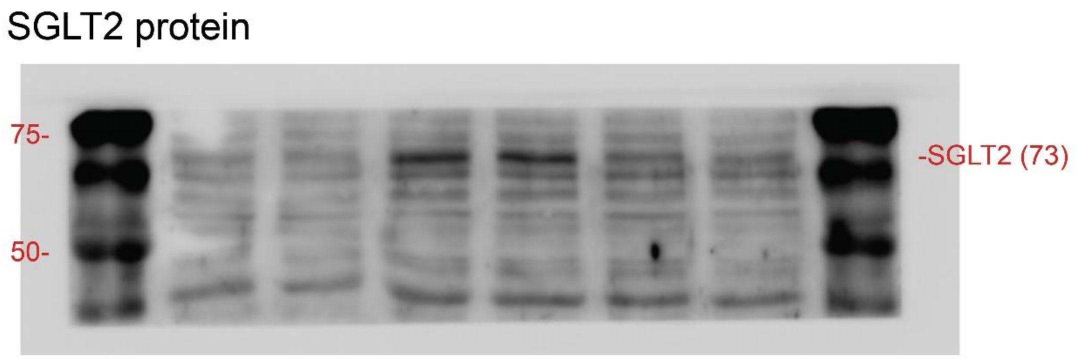

Fig S1. SGLT2 protein is expressed in human atrial fibroblasts. Western blot experiment was conducted for the detection of SGLT2 protein expression. Control atrial fibroblasts were lysed in radioimmunoprecipitation assay buffer containing 150 mmol/L NaCl, Nonidet P P40, 50 mmol/L Tris pH 7.4, 0.5% sodium deoxycholate, 0.1% sodium dodecyl sulfate (SDS) and protease inhibitor cocktails (Sigma-Aldrich). The proteins were fractionated using 10% SDS-polyacrylamide gel electrophoresis and transferred onto an equilibrated polyvinylidene difluoride membrane (Amersham Biosciences, Buckinghamshire, UK). Fractionated protein was probed with primary antibodies against Sodium-glucose co-transporter 2 (1:1000, polyclonal, Abcam), followed by incubation with secondary antibodies conjugated with horseradish peroxidase. Bound antibodies were detected using an enhanced chemiluminescence detection system (Millipore, Darmstadt, Germany) and analyzed using AlphaEaseFC software (Alpha Innotech, San Leandro, CA, USA).
